# The learning curve on uniportal video-assisted thoracoscopic lobectomy with the help of postoperative review of videos

**DOI:** 10.3389/fonc.2023.1085634

**Published:** 2023-03-16

**Authors:** Zuodong Song, Yu Yuan, Chao Cheng, Qingquan Luo, Xinghua Cheng

**Affiliations:** ^1^ Shanghai Lung Cancer Center, Shanghai Chest Hospital, Shanghai Jiao Tong University, Shanghai, China; ^2^ Department of Thoracic Surgery, Dazhou Central Hospital, Sichuan, China; ^3^ Department of Thoracic Surgery, West China Hospital, Sichuan, China

**Keywords:** uniportal video-assisted thoracoscopic lobectomy, learning curves, review of videos, efficacy, proficiency

## Abstract

**Objectives:**

Video-assisted thoracoscopic lobectomy has become the preferred surgical approach in experienced centers, and uniportal approaches are becoming increasingly used. But the uniportal approach is still not widely applied presumably due to the learning difficulties of this complex procedure. The use of surgical videos may be helpful to accelerate the learning of this new techniques as in other fields. In this study, we aimed to analyze the learning curve of uniportal video-assisted thoracoscopic lobectomy with the help of postoperative review of videos.

**Methods:**

114 patients with early-stage lung cancer who underwent uniportal video-assisted thoracoscopic lobectomy performed from 2020 to 2021 were reviewed in this study. We recorded the operation video for each patient and reviewed all the videos after surgery. The learning curves were assessed using cumulative sum analysis and the collected data of perioperative outcomes were assessed.

**Results:**

The CUMSUM curve showed its inflection points were around case 38 and 53. It was less compared with previous studies, which about 57–140 cases are needed to attain the proficient phase. The perioperative outcomes were similar in each phase, which included intraoperative blood loss (79.00 ± 26.70 *vs* 70.67 ± 26.64 *vs* 70.56 ± 27.23, p=0.0119), the length of hospital stay (3.60 ± 1.52 days *vs*. 3.23 ± 0.90 days *vs*. 3.06 ± 0.88 days, p=0.053), the rate of prolonged air leak and conversion to open thoracotomy. There was also no significant difference in the numbers and station of lymph node dissection among the three phases.

**Conclusions:**

Uniportal video-assisted thoracoscopic lobectomy is a safe and reliable approach. Recording and reviewing the operation video could help the surgeon to improve deficiencies and refine the procedure.

## Introduction

With the full implementation of screening and the development of high-resolution computed tomography, the detection rate of early stage lung cancer has significantly increased (1). Minimally invasive surgery, which includes video-assisted thoracoscopic surgery (VATS), has become the preferred approach for the curative treatment of early stage lung cancer (2, 3).

In the last decade, VATS lobectomy has become the preferred surgical approach in experienced centers, and is usually performed through 2–4 ports. Uniportal VATS (U-VATS) is based on the conventional VATS with reduced auxiliary operation ports. However, the U-VATS technique is still not widely applied in most medical centers, presumably due to the learning difficulties of this complex procedure (4–8).

Video review has been proved to be a useful tool for learning new skills in many fields such as athletics, modern drama and aviation. The use of surgical videos is also emerging as a powerful tool to facilitate the acquisition of new surgical skills and to accelerate the learning of new techniques (9, 10). However, these studies are usually limited to the study of multiportal laparoscopic technology and there is no research report on the uniportal thoracoscopy technology. In this study, we aimed to describe our experience in 114 consecutive cases and to analyze the learning curve of uniportal video-assisted thoracoscopic lobectomy with the help of video-assisted operative feedback.

## Materials and methods

### Patients

This retrospective study was reviewed and approved by the ethics committee of the Shanghai Chest Hospital. All patients have signed the written informed consent before the operation. All operations were performed by the same surgeon (Dr. Xinghua Cheng) from Shanghai Chest Hospital, which had performed 88 cases of multiportal thoracoscopic lobectomy before. Totally 114 consecutive patients who underwent uniportal VATS lobectomy from May 2020 to August 2021 were reviewed in this study.

### Surgical technique

The patients were maintained in the lateral decubitus position and 1-lung ventilated with double-lumen endotracheal intubation, received general anesthesia. The surgeon was on the patient’s abdominal side, and the assistant was on the opposite side (the back of the patient). A 2.5 to 3 cm hole was made at the fifth intercostal space on the middle axillary line. Wound protectors were used at the incision to facilitate exposure and simplify instrumentation.

The camera was positioned on the posterior portion of the incision. The staplers were always introduced through the most anterior portion of the incision, below any other instrument, and fissures were always cut with energy sealing devices. The bronchus, vein and artery were divided anatomically, and dissected separately using endoscopic staplers or ligated by using hem-o-locks before dissection. 24 Fr chest tubes were inserted through the incision at the end of the operations, and we would remove the chest tube if the patient’s volume of drainage was less than 200ml per day and there was no air leakage.

### Video review of surgical skills

We recorded the operation video for each patient and reviewed the videos after surgery. The video recording was started with the introduction of a dissector and concluded with the removal of the target lobe and lymph nodes. All videos were assessed from three domains of bimanual dexterity, efficiency and tissue handling based on the surgical performance. Besides, all the operation videos were analyzed for frequency of minor technical errors and adverse events after surgery. Minor errors included insufficient exposure, wrong pass angle of cutting stapler, dropping tissue or suture. Examples of adverse events included excessive blood loss, tears of lung or bronchus requiring repair. Meeting quarterly, we reviewed the ‘‘typical’’ and ‘‘challenging’’ operation videos with senior surgeons to share best practices and identify where the technique could be improved.

### Data collection

All patients were characterized by demographic and clinical variables, including sex, age, smoking history, body mass index (BMI), Charlson comorbidity index (CCI), forced expiratory volume in 1 s (FEV1), diffusion capacity of the lung for carbon monoxide (DLCO), pathology, tumor size, operation procedure, and lymph node (LN) status. Surgical outcomes included procedure time, intraoperative blood loss, postoperative hospital stay, complications, and lymph node retrieval. Procedure time was defined as the time from the first incision to complete closure of the skin. Prolonged air leakage was defined as air leakage lasting for >5 days postoperatively. Perioperative mortality included death during hospitalization or within the first 30 days after the operation.

### Statistical analysis

Statistical analysis was performed using SPSS software (version 18.0; SPSS, Inc., Chicago, IL) and R (version 3.6.2). Data are shown as mean (standard deviation) or median (interquartile range) for continuous variables and as n (%) for categorical variables. Differences between groups were analyzed using one-way analysis of variance or the Kruskal-Wallis *H* test. Fisher’s exact test or chi-square test was used to classify variables. A two-sided P-value < 0.05 was considered statistically significant. The cumulative sum (CUSUM) analysis method was used to quantitatively assess the learning curve. CUSUM for operation time was calculated as follows: $CUSUM=\sum_{i=0}^{n}\left(x_{i}-u\right)$ , where *x_i_* and *u* respectively represent an individual and the mean overall operative time (6). In addition, we established a polynomial trend line to show the change in the slope of the learning curve. According to the learning curve obtained from the analysis, we divided it into three stages: the ascending phase (Phase I), the transition phase (Phase II), and the maturity phase (Phase III).

## Results

### Patient characteristics

Altogether, 114 patients who undergoing U-VATS lobectomy performed by a single surgeon between May 2020 and August 2021 were enrolled in this study. The baseline characteristics of the patients are shown in **Table 1**. Of the 114 patients, 50(43.86%) were men and 64(56.14%) were women. The median age of the patients was 61 years. Adenocarcinoma was the most frequent histologic type(87 patients, 76.3%), and the mean tumor diameter was 21.00 ± 12.80 mm. Detailed patient characteristics are presented in **Table 1**. When clinical demographics and characteristics were assessed for the three periods of the learning curve, there were no significant differences between patients in each learning period.

**Table 1:** Patients’ demographics and surgical outcomes according to learning curve phases.

Parameters	Total (n=114)	Phase I (n=35)	Phase II (n=18)	Phase III (n=61)	*P*
Patients number		1-35	36-53	54-114	
Gender, n (%)					
Male	50 (43.86%)	11 (36.7%)	13 (43.3%)	26 (48.15%)	0.113
Female	64 (56.14%)	19 (63.3%)	17 (56.7%)	28 (51.85%)	
Age, year					0.721
Mean ± SD	59.82 ± 9.99	59.73 ± 10.86	60.03 ± 8.82	59.74 ± 10.27	
BMI					0.553
Mean ± SD	23.16 ± 3.05	23.73 ± 3.00	22.62 ± 2.65	23.14 ± 3.27	
CCI					0.717
Median(IQR)	2.0(2.0-3.0)	2.0(2.0-3.0)	2.0(2.0-3.0)	2.0(2.0-3.0)	
FEV1(%)					0.348
Mean ± SD	98.48 ± 13.63	97.92 ± 13.50	97.80 ± 11.46	99.16 ± 14.95	
DLCO(%)					0.362
Mean ± SD	93.82 ± 17.33	93.54 ± 16.21	92.89 ± 17.72	94.48 ± 17.99	
Smoking history (%)	38(33.3%)	12(40%)	11(36.7%)	15(27.8%)	0.080
Pathology					0.291
Adenocarcinoma	87(76.3%)	21(70%)	23(76.7%)	42(77.8%)	
Squamous	11(9.6%)	4(13.3%)	3(10%)	6(11.1%)	
Others	16(14%)	5(16.7%)	4(13.3%)	6(11.1%)	
Tumor size					0.094
Mean ± SD	21.00 ± 12.80	21.93 ± 12.15	22.53 ± 14.78	19.63 ± 12.06	
Operation procedure					0.073
RUL	45(39.5%)	16(53.3%)	10(33.4%)	20(37%)	
RML	12(10.5%)	3(10%)	4(13.3%)	5(9.3%)	
RLL	21(18.4%)	7(23.3%)	6(20%)	8(14.8%)	
LUL	19(16.7%)	2(6.7%)	6(20%)	10(18.5%)	
LLL	17(14.9%)	2(6.7%)	4(13.3%)	11(20.4%)	
Operative time, min					<0.001
Mean ± SD	86.09 ± 21.23	99.40 ± 18.72	85.93 ± 20.93	78.78 ± 19.36	
Blood loss					0.119
Mean ± SD	72.81 ± 26.96	79.00 ± 26.70	70.67 ± 26.64	70.56 ± 27.23	
LN stations					0.521
Mean ± SD	5.82 ± 1.68	5.93 ± 1.98	5.67 ± 1.37	5.85 ± 1.68	
LN numbers					0.086
Mean ± SD	8.23 ± 3.63	7.93 ± 3.82	7.63 ± 2.86	8.72 ± 3.89	
Length of stay, days					0.053
Mean ± SD	3.25 ± 1.10	3.60 ± 1.52	3.23 ± 0.90	3.06 ± 0.88	
Prolonged air leak (%)	4(3.5%)	2(6.7%)	0	2(3.7%)	0.098
Conversion to open thoracotomy (%)	2(1.8%)	1(3.3%)	0	1(1.9%)	0.177

BMI, Body mass index; CCI, Charlson comorbidity index; FEV1%, forced expiratory volume in 1 second to forced vital capacity ratio; DLCO%, Diffusion capacity of the lung for carbon monoxide ratio; RUL, right upper lobe; RML, right middle lobe; RLL, right lower lobe; LUL, left upper lobe; LLL, left lower lobe; LN, Lymph node.

### Learning curve analysis

**Figure 1:**
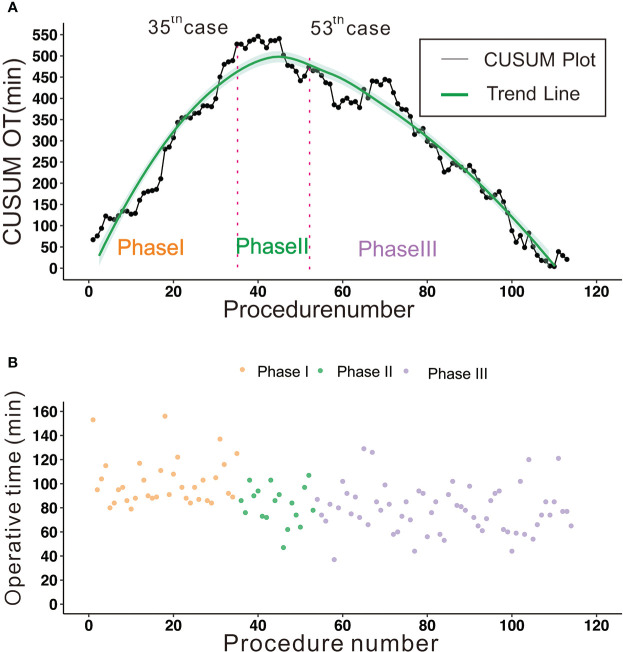
**(A)** The CUSUM curve of operative time; 35 cases were needed to lay the technical foundation and 53 cases were necessary to achieve proficiency. Phase I: 1-35 cases, learning phase. Phase II: 36-53 cases, transition phase. Phase III: 54-114 cases, proficiency phase. **(B)** The raw operative times were plotted in chronologic surgery order.

The raw operative time for all 114 patients is shown in **Figure 1B**. As the number of procedures increased, the operation time decreased and became stable eventually. The learning curve for operative time is shown in **Figure 1A**. According to the trend and inflection points of the curve, we obtained three well-differentiated phases: phase I (1–35 cases), phase II (36–53 cases), and phase III (54–114 cases). Phase I was the ascending slope of the curve, which represented the initial experience of the technique learning, and Phase II was the transition part of the curve, which represented further improvement in surgical skills. Phase III was the descending slope, which indicated that proficiency had been achieved.

### Perioperative outcomes and subgroup analysis

In the all patients, the mean operative time and length of stay were 86.09 ± 21.23 minutes and 3.25 ± 1.10 days (**Table 1**). No patient died perioperatively, and two cases were converted to open thoracotomy due to severe adhesions in thoracic cavity (1 case) and vascular accident (1 case). Only four patients (3.5%) experienced prolonged air leak after surgery.

**Figure 2:**
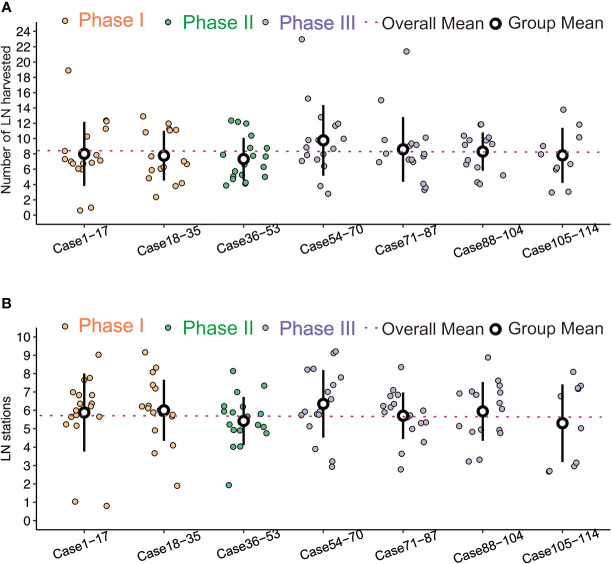
**(A)** Comparisons of blood loss, no significant difference between the three phases. **(B)** Comparisons of postoperative stay, no significant difference between the three phases.

**Figure 3:**
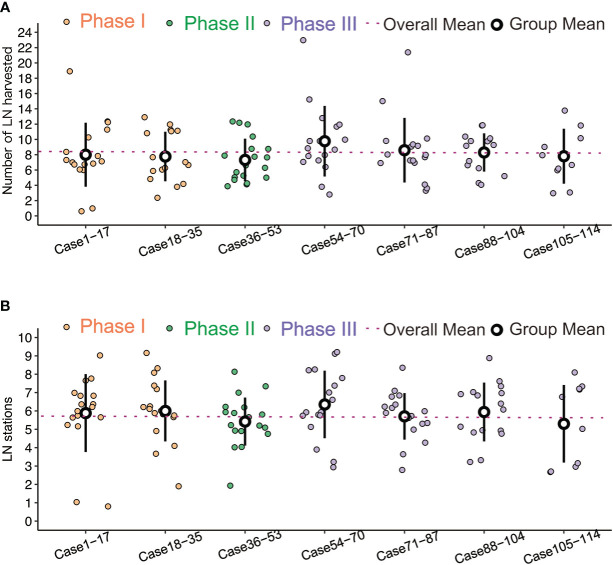
**(A)** Comparisons of LN harvested number, no significant difference between the three phases. **(B)** Comparisons of LN station, no significant difference between the three phases.

In the subgroup analysis, the operative time improved from a mean of 99.40 ± 18.72 minutes to 78.78 ± 19.36 minutes, with a significant difference (p<0.001). Intraoperative blood loss tended to decrease, but there was no significant difference between the three phases (P = 0.119) (**Figure 2A**). The length of hospital stay was reduced (3.60 ± 1.52 days *vs*. 3.23 ± 0.90 days *vs*. 3.06 ± 0.88 days), but this was not statistically significant (P = 0.053) (**Figure 2B**). There was no significant difference in the stations (p=0.521) or numbers (p=0.086) of lymph node dissection among the three phases (**Figure 3**).

## Discussion

U-VATS lobectomy has been proven to be a safe and feasible surgical approach for early stage lung cancer (11–13). However, it is not widely applied in most medical centers because of technical difficulties. Currently, the use of surgical videos by surgeons facilitate the learning of new procedures and techniques (9, 10). Surgical videos could record the frequency of minor technical errors and adverse events. Surgeons can review these videos to continuously reduce and correct these errors, thereby reducing the incidence rate of potential patients’ morbidity. There is always a sharp contrast between what the surgeons think they did and what actually happened, postoperative review of videos would be helpful for operators to addresses important cognitive limitations (14–17). Besides, reviewing the ‘‘typical’’ and ‘‘challenging’’ operation videos with senior surgeons allows accurate assessment and identifying where the technique could be improved. All of the above can help improve surgical techniques, thus to accelerate the learning curve of surgeons and improve the surgical safety. Nevertheless, most of the research on video learning is limited to laparoscopic technology, there is few research focus on the thoracoscopy technology, especially on the uniportal thoracoscopy technology.

In the present study, we analyzed 114 cases of U-VATS lobectomy performed by a single surgeon using the CUSUM method to evaluate how video review promotes the learning curve. It took 35 consecutive cases of U-VATS lobectomy to complete the ascending phase and 18 additional cases to overcome the transition phase. In other words, efficacy was achieved after 35 cases and proficiency was achieved after 53 cases. No patient died preoperatively, regardless of the phase of the learning curve. There was no significant difference between the three phases in terms of both intraoperative blood loss and length of hospital stay. There was also no significant difference in the number and station of lymph node dissection among the three phases. These results indicate that U-VATS lobectomy is safe and reliable, even during the initial phase of learning the technique.

So far, studies have been carried out in large-volume centers to analyze the learning curve of U-VATS lobectomy regardless of video review (**Table 2**) (7, 18–20). According to the results of these study, about 57–140 cases are needed to attain the proficient phase. Compared with our results, this number is obviously higher than required, which may indicate that video review can accelerate the learning curve of surgeons. Moreover, almost all previous studies showed that the amount of blood loss and length of hospital stay showed a downward trend among the three distinct periods of the learning curve. In other words, the other surgeons’ operations were not sufficiently stable at the beginning of the learning curve. However, in our study, all the perioperative outcomes of patients in the three phases were comparable. This may be what was caused by the earlier mentioned video review that can help reduce intraoperative errors and improve surgical safety. Besides, in some of these previous studies (7), there was significant difference in the numbers or stations of lymph node removed because of performing lymph node dissection through the uniportal technique remains challenging. But there was no significant difference in the numbers and stations of lymph node dissection among the three phases of our study. The comparative analysis of these research results all suggest that video review can help surgeons learn uniportal thoracoscopy more quickly and safely.

**Table 2:** Surgical outcomes of previous U-VATS research.

Author, year, country	Number of patients included	Number of cases needed to get the proficient phase	Mean or median operation time (min)
Xiaochuan Liu et al. (18), China	120	61	Phase1 92.1 ± 20.7 Phase2 65.9 ± 22.5 Phase3 52.6 ± 10.4
Liang Chen et al. 2020 (19), China	124	57	Phase1 130 Phase2 110 Phase3 105
Shenghui Li et al. (20), China	397	71	Phase1 140 Phase2 123 Phase3 116
Arthur Vieira et al. (7), Canada	274	140	Phase1 158.8± 52.2 Phase2 145.9± 43.8 Phase3 117.9 ± 32.6

Video review is associated with shortened learning curve and reduced intraoperative accidents such as bleeding probably implicates accelerated self-improvement of surgical skills. It is particularly important when new technique is to be implemented or to be transferred to new trainees. By recording surgical videos, surgeons can not only review by themselves but also compare the videos with the operations from more experienced surgeons, and ask for suggestion from senior peers. For intraoperative accidents, it is easier for the surgeon to re-think how to confound and avoid similar situations after the surgery. Besides, a surgical video database is important for surgical education and generate future artificial intelligence guided surgical programs (21, 22). Video review is very practical and eliminates many inconveniences and risks associated with on-site surgical guidance. It can also be of great help in the training and education of surgical residents (23). The emergence of complex thoracoscopic and robotic surgeries transfers surgical experience from junior residents to more advanced trainees. Intraoperative learning is further limited by increasing concerns for patient safety and the possibility that resident teaching may prolong surgery time. These challenges indicate a need for innovative educational strategies to maximize the learning of operative skills (24). The electronic transmission of video is well suited for remote viewing without the limitations of location and environment. Surgical residents can use fragmented time to conduct video review and analysis freely. In addition, they can share videos with peers or more experienced surgeons to obtain feedback. Nowadays, most thoracoscopic equipment comes with video recording capability, making video recording of surgeries very easy. With the fast development of imaging and AI technologies, video review methods will become an increasingly valuable tool to accelerate innovation and promote safer surgeries.

Despite our best efforts, this study has some limitations. First, this study was a retrospective study, in which the selection bias is inevitable. Second, the residents, fellows, and nursing teams were not the same for every procedure. There was no definite evidence to demonstrate whether these could have impacted the learning curve. Third, there was no clear definition of a ‘‘learning curve’’ for this procedure, and there were varying definitions of proficiency (25–27).

## Conclusion

In conclusion, uniportal VATS lobectomy is a safe and reliable approach, and the surgeon with the help of postoperative review of videos is better able to improve deficiencies and can better refine the procedure. In the results of our study, with the help of video review, efficacy was reached after 35 cases, and proficiency was achieved after 53 cases.

## Data availability statement

The original contributions presented in the study are included in the article/supplementary material. Further inquiries can be directed to the corresponding author.

## Ethics statement

The studies involving human participants were reviewed and approved by the ethics committee of the Shanghai Chest Hospital. The patients/participants provided their written informed consent to participate in this study.

## Author contributions

XC and QL conceived and designed the study. ZS and YY wrote the paper. CC performed data analysis. ZS and XC reviewed and edited the manuscript. All authors contributed to the article and approved the submitted version.
